# Yuan Longping and Hybrid Rice Research

**DOI:** 10.1186/s12284-021-00542-4

**Published:** 2021-12-13

**Authors:** Qian Qian, Fan Zhang, Yeyun Xin

**Affiliations:** 1 Institute of Crop Sciences, Chinese Academy of Agricultural Sciences, Beijing, 100081 China; 2 China National Hybrid Rice Research and Development Center, Changsha, 410125 China

On May 22, 2021, Mr. Yuan Longping, academician of Chinese Academy of Engineering and winner of the Medal of the Republic, passed away. Professor Yuan has been known as a renowned rice breeder, world hunger fighter and the father of hybrid rice. We knew Professor Yuan when we were young. He gave us many supervisions and directions, often encouraging us to make scientific innovations, which was Professor Yuan’s spirit and also a permanently pursuing thing in his lifetime. It is forefathers like Professor Yuan who had taken great efforts to ensure that we eventually get enough to eat. His merits in scientific research and hybrid rice development will be ever remembered in the annals of history. It is a great honor and privilege for us to dedicate this article to summarize Professor Yuan’s special contribution to developing hybrid rice and fighting the future world hunger.

In the past 50 years, Professor Yuan had endured life hardship, persevered in his pursuit, innovated scientific research, and finally climbed the peak in hybrid rice research and development. With his scientific research and contribution, China has reached the forefront of worldwide in hybrid rice research, development and application. He not only improved China’s food self-sufficiency but also significantly contributed to the world food security through technical extension and commercialization of hybrid rice. The following four aspects highlight his accomplishments and contributions.

First, Professor Yuan was the pioneer in hybrid rice research and development. In 1964, Professor Yuan broke the bonds of the prevailing genetic dogma at that era that should be no significant heterosis in rice because it is self-pollinated, and started his research on three-line hybrid rice in China. The landmark paper he published, “Male Sterility in Rice”, proposed that to utilize rice heterosis, male sterility should be used first as large-scale hand emasculation is extremely difficult. During the 1970s, he supervised the China National Cooperative Research on Hybrid Rice and proposed a novel technical approach of “distant hybridization between wild rice and cultivated rice” to create male-sterile lines. In 1972, together with his assistant, he developed the first set of male-sterile and maintainer lines, Er-jiu-nan-1 A and B. The next year, the restorer line was identified to complete the “three-line” system and develop the first high-yielding commercial rice hybrid, Nanyou 2. Hybrid rice breeding and production became widespread in China by 1976. Public hybrid rice commercialization in China led to a substantial increase in rice production, improving both the yield per hectare and total production dramatically. In general, hybrid rice outyields the conventional rice by 20–30%. To date, over 6 billion ha of hybrid rice have been grown in China, increasing total accumulated rice yield by over 0.6 billion tons. Recently, hybrid rice area in China has increased to over 16 million ha per year, accounting for 57% of total rice areas and approximately 65% of total rice production. Commercial hybrid rice has increased the yield by about 2.5 million tons per year, which can feed an additional 80 million people, achieving the target at grain self-sufficiency in China and also demonstrating China’s role in advancing agricultural technology.

Second, Prof. Yuan contributed to continuous development and transformation of hybrid rice. In 1986, he proposed a strategic technical plan for three phases of hybrid rice breeding and development, based on three-line or CMS (cytoplasmic male sterile) system, two-line or P(T)GMS (photo-sensitive or thermosensitive genic male sterility) line system, and one-line or apomixis system, making the breeding program becomes simpler and more efficient. The three strategic phases represent the utilization of heterosis at three levels: intervariety, intersubspecies, and interspecies, profiling scenarios of increased heterosis utilization. According to his proposal, each phase of hybrid rice development will lift the ceiling of rice yield to a significantly higher level. This strategic concept outlines the direction for long-term hybrid rice research and development. The two-line hybrid rice project was adopted by Chinese National Hi-Tech Plan in 1987, and Professor Yuan served as chief scientist to supervise the national cooperation. At this position, he proposed a series of technical improvements, including determination of suitable temperature for the fertility conversion of photo-thermo-sensitive genic male-sterile lines, core seed production procedures, breeding and selection through irrigation with cold water, and many other technologies. Cooperative breeders were organized into special working groups to overcome a series of technical challenges, and eventually two-line hybrid rice was successfully developed. The concept development and commercialization of two-line hybrid rice is a Chinese invention, representing a watershed moment in crop breeding. Commercialization of two-line hybrid rice demonstrates the expertise of Chinese hybrid rice experts, led by Professor Yuan, and ensures China standing at the forefront of global hybrid rice development.

To improve photosynthetic efficiency, Professor Yuan proposed in 1997 to develop super high-yield hybrid rice through morphological modeling and breeding methodology, and initiated the research and development project “Chinese Super Hybrid Rice”. He led his team to successfully solve all the critical challenges in developing super hybrid rice during the last twenty years, achieving yield targets of 700, 800, 900, and 1000 kg per mu (1 ha = 15 mu) in large-scale planting areas in 2000, 2004, 2011, and 2014, respectively. In particular, a new record of rice yield was created in Gejiu, Yunnan with an average of 1067 kg per mu (*i.e*. 16 tons/ha) in years 2015, 2016, and 2017, and 1134 kg (*i.e.* 17 tons/ha) for years 2018, 2019, and 2020. Furthermore, 1152.3 kg of rice per mu (*i.e*. 17.3 tons/ha), averaged from the 6.67-ha demonstration plot in 2018, was also a new yield record for large-scale commercial production. Simultaneously, he led the team to develop the third-generation hybrid rice by using the nuclear male sterile system, and the newly-released variety “Sanyou No. 1” is suitable for planting as late rice in double-cropping areas with a yield of 13.5 tons per ha. Furthermore, he widened his research and made a breakthrough in developing salt-tolerance hybrid rice. After a series of experiments along the beach areas (under the condition of soil salt concentration less than 0.3%), the selected hybrid yielded up to 7.5 tons per ha.

Prof. Yuan always attempted to transform hybrid rice production through integrated breeding and crop management practices. Since 2006, he had developed two concepts for accelerating the commercialization of newly developed super hybrid rice. The first is “Sow Three, Yield Four” high-yielding project, which produces total yield on FOUR mu (0.27 ha) land in the past but with THREE mu (0.20 ha) land using currently available advanced super hybrid rice technology. By 2016, this concept has been adopted by 51 counties (cities and districts), with a planting area of 0.588 million ha and a total yield increase of 1.1 million tons. Currently, this high-yielding project has been undertaken by many rice-growing provinces, including Anhui, Henan, Guangdong, Guangxi, Yunnan and Guizhou. The second concept is associated with the “Three One” project, which is to feed THREE persons using the rice grain produced on ONE mu (0.067 ha) arable land with 360 kg of rice grain per year for a person. This concept has been demonstrated in Hunan for many years, with multiple modes and promising results achieved, through double-cropping rice, one-season super hybrid rice plus ratoon rice, one-season super hybrid rice plus potato, etc*.* Particularly in the double-cropping rice demonstration, annual yield per ha can be increased up to 18 tons. With the breeding success in developing the third-generation hybrid rice, particularly since the release of “Sanyou No. 1”, hybrid rice has become increasingly popular as late rice in the double-cropping areas. Therefore, Professor Yuan proposed the “1500 kg Project” in 2020, and carried out key research demonstrations with the goal of annual yield of 1500 kg per mu (22.5 tons per ha) in double-cropping areas in 2020 and 2021, using the most recently developed third-generation hybrid rice. In 2020, “Sanyou No. 1” as late rice in the demonstration field in Hengnan county, Hunan province, produced 13.67 tons per ha, while it yielded 9.29 tons per ha as early rice. As a result, the double-cropping rice produced the target annual yield of 22.96 tons per ha. The replicated trial in 2021 achieved the target results again, with annual yield of 24.06 tons per ha (14.04 tons per ha for late rice and 10.02 tons per ha for early rice). This is another significant accomplishment for double-cropping rice.

Third, Professor Yuan improved the world food security by devoting his life to “developing hybrid rice to benefit the people of the world”. To fulfill his great desire, he had been working for many years to make hybrid rice adopted and commercialized internationally, with particular contributions to scientific and technological exchanges, technical training, and foreign aid cooperation at the international level. He was invited by Food and Agriculture Organization (FAO) to be the chief consultant on international hybrid rice projects, and share his knowledge, experience, ideas and valuable breeding materials with scientists outside China to help develop hybrid rice to alleviate food shortage and hunger. He visited the International Rice Research Institute (IRRI) 30 times, and more than ten times to rice-growing countries including India, Vietnam, Myanmar, the Philippines, and Bangladesh. Since 1980s, Professor Yuan and his colleagues have run over 140 international training courses on hybrid rice in China for 14,000 government officials and agricultural scientists from over 80 countries. The trainees have become the technical backbone for local research and hybrid rice development after their return. With technical assistance from China and IRRI, India, Bangladesh, Indonesia, Vietnam, the Philippines, the United States, and Brazil have become large-scale hybrid rice growing countries, with seven million hectares planted in 2017.

Fourth, Professor Yuan established the discipline of hybrid rice. He has outstanding theoretical achievements to his credit with more than 60 papers and 7 monographs published. Among them, “Breeding and Cultivation of Hybrid Rice”, “Hybridriceology”, and “Breeding and Cultivation of Super Hybrid Rice” were awarded, respectively, the First Prize of the Best Science and Technology Books, the National Book Award, and the Chinese Government Award for Publishing. The book “Technology of Hybrid Rice Production” published by FAO has been distributed to over 40 countries as a guiding reference for hybrid rice research, extension and seed & grain production. Professor Yuan, as both an academic leader and a teacher, trained and supervised a large number of hybrid rice experts and technical backbones, established a comprehensive and integrated set of hybrid rice theory and production technology, and thus created a systematic and new discipline, Hybrid Rice Science.
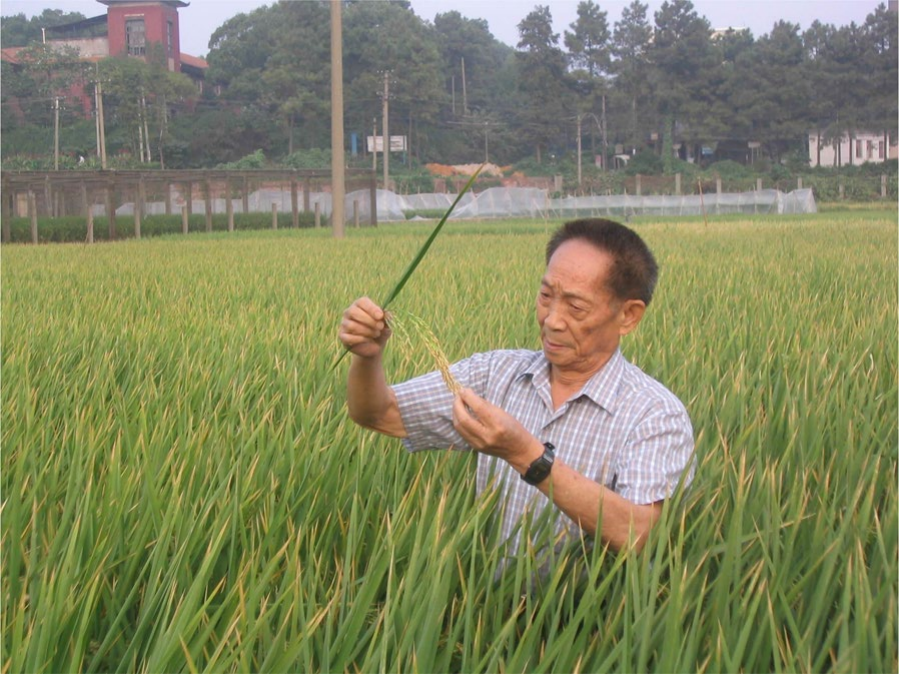


Prof. Yuan Longping (1930–2021).

Photo taken on Sep. 26, 2005 By Yeyun Xin.

## Data Availability

Not applicable.

## Abbreviations

CMS: Cytoplasmic male sterile
P(T)GMS: Photo-sensitive or thermosensitive genic male sterility
FAO: Food and agriculture organization

